# Aggressive Sternum Extension in Diffuse Large B-Cell Lymphoma: Barriers to Timely Care in a Resource-Limited Setting

**DOI:** 10.1155/crh/4886353

**Published:** 2025-12-04

**Authors:** Anahí Morales-Pedraza, Zulia Guzmán-Martínez, Ana C. Tejada-Vásquez, Héctor A. Vaquera-Alfaro, Silvia E. Haces-Rodríguez, Alia Guadalupe Ordoñez-Ayala, Perla R. Colunga-Pedraza

**Affiliations:** ^1^Servicio de Hematología, Hospital Universitario “Dr. José Eleuterio González”, Universidad Autónoma de Nuevo León, Monterrey, Mexico; ^2^School of Medicine, Universidad Autónoma de Nuevo León, Monterrey, Mexico; ^3^Department of Radiation Oncology, Hospital Universitario “Dr. José Eleuterio González”, Universidad Autónoma de Nuevo León, Monterrey, Mexico

**Keywords:** aggressive lymphomas, patient delay, patient interval

## Abstract

Non-Hodgkin lymphoma englobes a diverse group of malignant disorders. Although most commonly manifested as lymphadenopathies or solid tumors, some lymphomas can exhibit highly aggressive behavior, such as diffuse large B-cell lymphoma (DLBCL). This report highlights the case of a 72-year-old male from a resource-limited setting who delayed seeking medical care for two years, relying on alternative medicine for a destructive sternal mass. In low- and middle-income countries (LMICs), healthcare-seeking decisions are influenced by factors such as poor dimension of symptoms, cultural beliefs, limited access to health care, and reliance on traditional, conventional, and alternative treatment. This case highlights challenges in LMICs in cancer care and the urgent need to address these barriers. This case proposes that efforts should focus on reducing patient intervals and improving cancer outcomes in LMICs.

**Summary**

• Aggressive lymphomas progress rapidly, requiring prompt medical intervention to avoid severe outcomes, especially in resource-limited places.
• Patient interval is influenced by lack of awareness, cultural beliefs, use of traditional, conventional, and alternative medicine, financial toxicity, and fragmented healthcare systems.
• Efforts should be made to improve accessible cancer care.

## 1. Introduction

Aggressive lymphomas, like diffuse large B-cell lymphoma (DLBCL), are challenging due to their rapid progression and potential for life-threatening complications if untreated. Even a short delay in seeking medical attention can have serious consequences. Factors involved in the delay of attention, particularly for patients living in underprivileged circumstances in Latin America, comprise lack of awareness, the high costs of cancer care, a fragmented health system, and cultural beliefs that include the reliance in complementary or alternative medicine [1].

This case report describes a recent case of an aggressive DLBCL in an elderly male living in a constrained resource setting, initially treated with alternative medicine. Despite these interventions, the tumor persisted and progressed, leading to significant sternal engagement marked by destructive growth and the development of an exophytic necrotic sternal mass.

## 2. Case History

**Figure 1:**
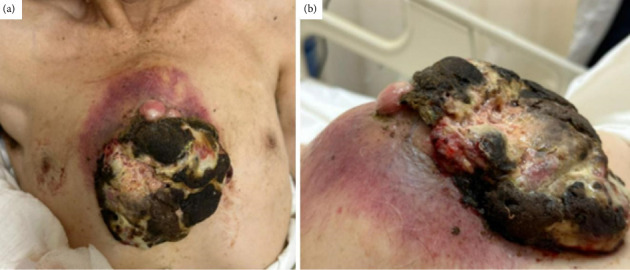
Clinical presentation. (a and b) Mass affecting the sternum and anterior thorax, with visible necrosis and surrounding erythema. *Source: Original work.*

A 72-year-old Hispanic male presented to the emergency department of our hospital with a sternal mass evolving over two years, previously managed unsuccessfully with unspecified homeopathic therapies. Upon admission, the patient exhibited a compromised performance status (ECOG 3, Karnofsky 60%). The physical examination revealed a prominent exophytic necrotic sternal mass (Figure 1) of approximately six intercostal spaces in size, accompanied by a palpable 3 × 3 cm axillary lymphadenopathy. The patient denied experiencing weight loss, night sweats, or fever. The patient was unemployed and had no health insurance; he lived in a low-income household of five-residents, all supported by his son, located more than 2 hours away from the hospital. He had a history of heavy smoking, alcoholism, and a recent diagnosis of arterial hypertension.

**Figure 2:**
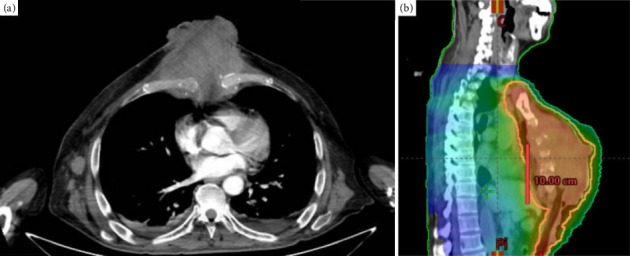
Imaging. (a) Transversal CT scan of the thorax. (b) Sagittal CT scan of the thorax showing planned radiotherapy target areas. *Source: Original work.*

At the admission, CT imaging was requested as soon as possible; however, it took 3 days to be performed. The study showed an irregular, heterogeneous mass in the anterior mediastinum with sternum bone resorption, presenting as an exophytic heterogeneous parasternal mass (Figure 2). Due to the extensive mass tissue and inaccessibility, the anterior chest wall tru-cut biopsy was delayed to 1 week after his admission and the results were obtained 3 days later, revealing a non-Hodgkin B-cell lymphoma. Immunohistochemistry staining demonstrated positive results for CD20, CD10, CD45, and MUM1, while CK Oscar and CD128 were negative.

The final diagnosis was DLBCL of the anterior mediastinum with aggressive extension into the anterior chest wall, involving the sternal bone, categorized as stage IV.

As the patient preferred palliative care over chemotherapy, local radiotherapy was initiated in the next three weeks as part of the palliative treatment, consisting of 12 sessions in total. Unfortunately, the patient discontinued follow-up due to financial and transport barriers and attempted to resume alternative medicine based on personal beliefs. This decision led to disease recurrence and systemic complications, ultimately resulting in the patient's demise 15 months after the diagnosis.

## 3. Discussion

This patient had a dramatic presentation of the disease, characterized by mass destruction of the sternum and part of the costal arch. However, there were alarming signals of the disease, and psychosocial and socioeconomic factors may have determined the decision to attend a medical check-up.

The concept of the patient interval—previously termed patient delay to avoid connotations of blame—refers to the period between the onset of the first cancer symptom recognized by the patient and the initial medical consultation. This phenomenon is common across cancer types [2] and commonly linked to lack of awareness of the seriousness of the clinical manifestations by patients [3] and has a significant impact on overall survival [4]. In lymphoma, longer patient intervals have been associated with lower socioeconomic status and less aggressive disease [5]. However, as illustrated in this case, even when the disease burden is severe, patient beliefs can strongly influence health-related decisions. Moreover, socioeconomic barriers may further prevent timely medical attention, despite the presence of obvious and debilitating symptoms.

Blood cancer is frequently diagnosed in advanced stages, in part due to difficult diagnosis because of the nonspecific symptoms that lead to the normalization of symptoms and adjudication of the manifestations to other mild illnesses [6]. However, this differs in the scenario of more alarming or severe manifestations, as patients often seek help early [3]. Thus, vague manifestations delay medical attention, while severe symptoms are help-seeking sooner. Although the patient clearly exhibited signs of illness, he underestimated them, perhaps as a reflection that in resource-limited settings, health concerns easily become secondary to day-to-day struggles until they become overtly life-threatening.

Other factors involved in delayed medical attention are emotional barriers and negative beliefs about cancer, such as fear, embarrassment, anxiety, and concern about the disease [7]. Individuals from lower socioeconomic backgrounds are more likely to develop fearful beliefs about the disease [2]; potential reasons involve poor understanding of the disease and negative experiences among friends and acquaintances [8]. Our patient exhibited reluctance to the medical attention, the diagnosis, and the proposed treatment received, likely due to pessimistic beliefs about his condition and a self-described perception of a fatalistic outcome, and even when an accessible palliative strategy of radiation was developed with him, his family, and the medical team, the patient expressed concerns of his condition and situation being challenged imposed founded in religious beliefs.

Priorities and insufficient funds also affect patients with low socioeconomic status [9]. Financial toxicity describes the material and emotional distress experienced by oncological patients due to the cost of cancer care that arises not only from direct healthcare expenses, such as the cost of treatment, but also from indirect healthcare expenses like travel costs [10, 11]. In this case, living more than 2 hours away from the hospital and relying on a single source of income to support an entire family meant a great challenge for our patient—even though many treatments at our center are free, travel and accommodation expenses are a common barrier for seeking care in reference centers located far away from the patient's home.

In most LMICs, health insurance coverage excludes a portion of the population, and even among those with health coverage, patients usually cover a part of the treatment cost. Most cancer drugs are costly, and most cancer regimens require multiple medications, making the treatment unaffordable for the majority of patients. Consequently, patients in low- and middle-income countries (LMICs) often rely on personal savings, the sale of belongings, or bank loans to cover healthcare expense [12]. However, even these disruptive alternatives may be unattainable for individuals in severe economic hardship. In such circumstances, patients may forgo seeking care altogether—as in this case, where the patient expressed that he believed he was destined to endure the disease and chose not to further jeopardize his family's financial stability.

Our patient did not have healthcare insurance. Limited access to healthcare and dissatisfaction with the quality of medical attention may lead patients to use traditional, complementary, and alternative medicine (TCAM), as our patient preferred. Although TCAM is generally considered a support treatment, people in developing countries may view it as a primary treatment probably because of its affordability, accessibility, and the limited availability of conventional therapy [13]. However, relying exclusively on TCAM can lead to delays in or rejection of allopathic medical treatments [13, 14].

In this case, the patient's delayed attention was influenced by a series of factors, such as lack of awareness about the illness, absence of health insurance, and reliance on complementary medicine. A substantial portion of the patients in many LMICs have limited resources, which leads to a delay in medical care and limited access to certain tools and treatments. This restriction causes poorer outcomes, as patients may not receive adequate interventions. We aim to highlight how economic disparity affects our population and encourage the government and health institutions to implement strategies to reduce this gap.

This case report stimulates reconsideration of the factors behind delayed medical care and seeks to raise awareness about financial toxicity.

## Data Availability

Data sharing is not applicable as no new data are generated.
